# Pathophysiology of Demineralization, Part II: *Enamel White Spots, Cavitated Caries, and Bone Infection*

**DOI:** 10.1007/s11914-022-00723-0

**Published:** 2022-02-14

**Authors:** W. Eugene Roberts, Jonathan E. Mangum, Paul M. Schneider

**Affiliations:** 1 American Board of Orthodontics, Indiana University & Purdue University at Indianapolis, 8260 Skipjack Drive, Indianapolis, IN 46236 USA; 2 Translational Proteomics Laboratory, Department of Biochemistry and Pharmacology, Faculty of Medicine, Dentistry and Health Sciences, University of Melbourne, Corner Grattan Street and Royal Parade, Melbourne, Victoria 3010 Australia; 3 American Board of Orthodontics, Melbourne Dental School, University of Melbourne, 720 Swanston St, Melbourne, Victoria 3010 Australia

**Keywords:** Cost-effective, Healthcare, Wellness, Dental caries, White spot lesion, Remineralization

## Abstract

**Purpose of Review:**

Compare noninfectious (part I) to infectious (part II) demineralization of bones and teeth. Evaluate similarities and differences in the expression of hard tissue degradation for the two most common chronic demineralization diseases: osteoporosis and dental caries.

**Recent Findings:**

The physiology of demineralization is similar for the sterile skeleton compared to the septic dentition. Superimposing the pathologic variable of infection reveals a unique pathophysiology for dental caries.

**Summary:**

Mineralized tissues are compromised by microdamage, demineralization, and infection. Osseous tissues remodel (turnover) to maintain structural integrity, but the heavily loaded dentition does not turnover so it is ultimately at risk of collapse. A carious tooth is a potential vector for periapical infection that may be life-threatening. Insipient caries is initiated as a subsurface decalcification in enamel that is not detectable until a depth of ~400μm when it becomes visible as a white spot. Reliable detection and remineralization of invisible caries would advance cost-effective wellness worldwide.

## Introduction

Demineralization is the fundamental mechanism for loss of mineralized tissue [1–5]. The biomechanics of routine loading, parafunction, and aging results in accumulation of microdamage that compromises structural integrity. Bone remodels to repair microdamage, but teeth are exposed to irreversible degradation due to attrition, abrasion, erosion, and abfraction [1]. Decreased mass of bones and teeth risks structural failure due to atraumatic fractures or cavitated caries. The etiology of demineralization involves acid produced by clastic cells or microbic metabolism [2–5]. Acidic fluid bathing calcified tissues dissociates the mineral component of hydroxyapatite (HA). The process may be physiologic, environmental, or pathologic [1]. Noninfectious demineralization was addressed in part I of this review. Part II of this clinically oriented series focuses on pathology of infection superimposed on the physiology of mineralized tissues. The pathophysiologic emphasis is on the most prevalent pandemic disease in the world: dental caries [4–8].

## Infections

**Fig. 1:**
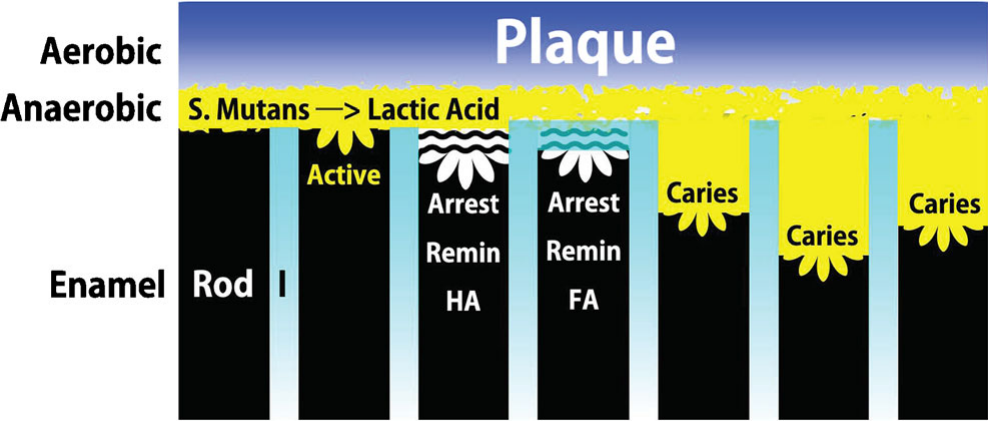
Based on the evidence reviewed, a schematic diagram of a plaque-coated enamel surface shows the principal stages of enamel pathophysiology relative to demineralization, remineralization and infectious dental caries. The superficial layer of plaque is aerobic, while the inner layer adjacent to the enamel surface is anaerobic. Enamel rods are separated by inter-rod substance (I) which has a more porous crystalline structure which allows percolation of fluoride to produce fluorapatite (FA). It is hypothesized that the inter-rod concentration of FA (cyan color gradient) is greatest near the oral surface and decreases with depth. Under anaerobic conditions, *S. Mutans* produces the lactic acid that facilitates active demineralization. Progressive caries preferentially demineralized the rods (HA prisms). From the left, black enamel rods depict stages of the pathophysiology: (1) intact rod, (2) active surface demineralization, (3) arrested lesion remineralized with HA, (4) arrested lesion remineralized with FA, and (5–7) active carious infection. See text for details

Lactic acid and any other organic acids [4, 5] produced by oral microbes tip the solubility equilibrium of HA toward dissolution for exposed dental tissues. The cariogenic microbes are nourished by the biofilm (plaque) that support a demineralization defect as it is formed [6–8]. Fluoride is a natural mineral in the environment that plays an important role in enamel physiology. Inter-rod substance (sheath) is more porous than the enamel rods. When there is optimal fluoride ion (F-) in the oral environment, the fluid component of saliva percolates through the enamel preferentially protecting the sheaths from demineralization by formation of fluorapatite (FA) via ion substitution during physiologic remineralization [1]. Thus, the enamel rods (HA prisms) are at greater risk of demineralization by the acidic challenge from active caries resulting in scooped-out deficits on the enamel surface (Fig. 1). Virulent microbes within plaque colonize the shallow defects (>10μm deep) to initiate an active carious lesion that is self-sustaining via fermentable carbohydrates in the diet [1]. The anti-caries effect of F^−^ is an ion exchange with hydroxyl (OH^−^) groups of HA to form FA. The biologic form of FA is a very stable, tight-packed apatite structure that is resistant to the acidic elevation of solubility equilibrium that would otherwise initiate enamel rod demineralization (Fig. 1) [1, 11].

Carious teeth are a potential vector for deep sepsis into bone that can result in painful and even life-threatening infections [1]. Pathophysiology is similar for dental and skeletal sepsis. Initial carious lesions in enamel (Fig. 1) are like infections in nonvital bone. Initial infection of dead bone, enamel, and cementum are all supported by microbe-laden biofilm [9–11]. Microbes must maintain an extravascular source of nutrition and waste disposal because there is no access to host vascularity. However, once the infection invades vital tissue such as dentin [4, 5, 12], the pathogenesis is similar to progressive infections in living bone [13]. The morphology of the lesions may be lytic, sclerotic, or a combination of the two [14]. Although dental caries may be associated with mechanical factors and chemicals, it is essentially an infection related to hygiene and diet [4–8]. Cementum exposure to the oral cavity increases with age as soft tissue recedes exposing the roots of teeth. The mineralized surface of a root (cementum) is more susceptible to plaque-induced caries than enamel because it is less densely mineralized and more susceptible to plaque retention [15].

The etiology and pathogenesis of biofilms (plaque) are best studied in sophisticated in vitro models [16]. The virulent bacteria in plaque are primarily *Streptococcus mutans (S. Mutans)* as well as numerous *Lactobacillus* species that metabolize dietary sugar to produce lactic acid. Epidemiology suggests that *S. mutans* is the predominant microbe, and its prevalence has more impact on caries risk than sugar consumption and poor oral hygiene [17]. Relative to the discussion of mineralized tissue infection, dental decay is clearly a plaque-dependent process which begins with pellicle, an acellular proteinaceous film formed by the saliva that coats all oral mineralized surfaces. Microbes attach to pellicle and form a biofilm which increases in volume to evolve into a dental plaque with a superficial aerobic zone and a deep anaerobic layer next to the enamel surface (Fig 1). In contrast to anaerobic bone infections [14], the cariogenic bacteria are nourished by the diet and particularly sugars that permeate the plaque. As will be discussed in detail in the subsequent caries section, the most virulent aspect of the disease process occurs in the anaerobic environment where *S. mutans* metabolism produces acid via fermentation. The decreased pH of plaque fluid attacks the enamel rods, cementum, and exposed dentin. As a facultative anaerobe, *S. mutans* produces adenosine triphosphate (ATP) by aerobic respiration if oxygen is present, but it is capable of switching to fermentation if oxygen is absent. *S. mutans* is naturally present in the human oral microbiota, along with at least 25 other species of oral streptococci, and each species has specific properties for colonizing in a specific niche [4, 5, 11, 12]. Although the disease process is well known, the prevention and treatment of dental caries is problematic. A few antimicrobial peptides display a remineralizing effect on HA [18], but hygiene and professional prophylaxis are the mainlines of defense. Caries is a communicable disease that must be inoculated with specific virulent bacteria [19].

If fermentable carbohydrates are consumed regularly, cariogenic bacteria may sufficiently compromise tooth and bone structure to produce serious bone infections. A particular concern is a fistula draining a periapical abscess into a body space like the floor of the mouth. In the absence of effective healthcare, body space infections are fatal. Prior to antibiotics, body space infections had a poor prognosis even in developed countries. In the era of modern antibiotics, odontogenic body space infections continue to be complex, life-threatening problems [20]. In a recent sample (*n*=256), a dental abscess origin was found for 76% of body space infections in the head. Despite surgical drainage and antibiotics, three were fatal [21•]. Ludwig’s angina with airway compromise continues to be a serious complication for uncontrolled caries infections [22]. An additional serious sequela for bone infections is septicemia which is usually associated with (1) compromised immune system [23], (2) resistant strains of bacteria due to misuse of antibiotics, and (3) failure to eliminate the source of the infection [24]. Caries is a serious health challenge. Furthermore, dental negligence is an invitation to serious medical problems worldwide [25, 26]. The Healthcare Effectiveness Data and Information Set (HEDIS) documents that compromised dental health is associated with poor adherence to preventive measures for cancer and retinopathy, as well as for glycemic and blood pressure control for potential diabetics [26]. Health and wellness are closely related.

**Fig. 2:**
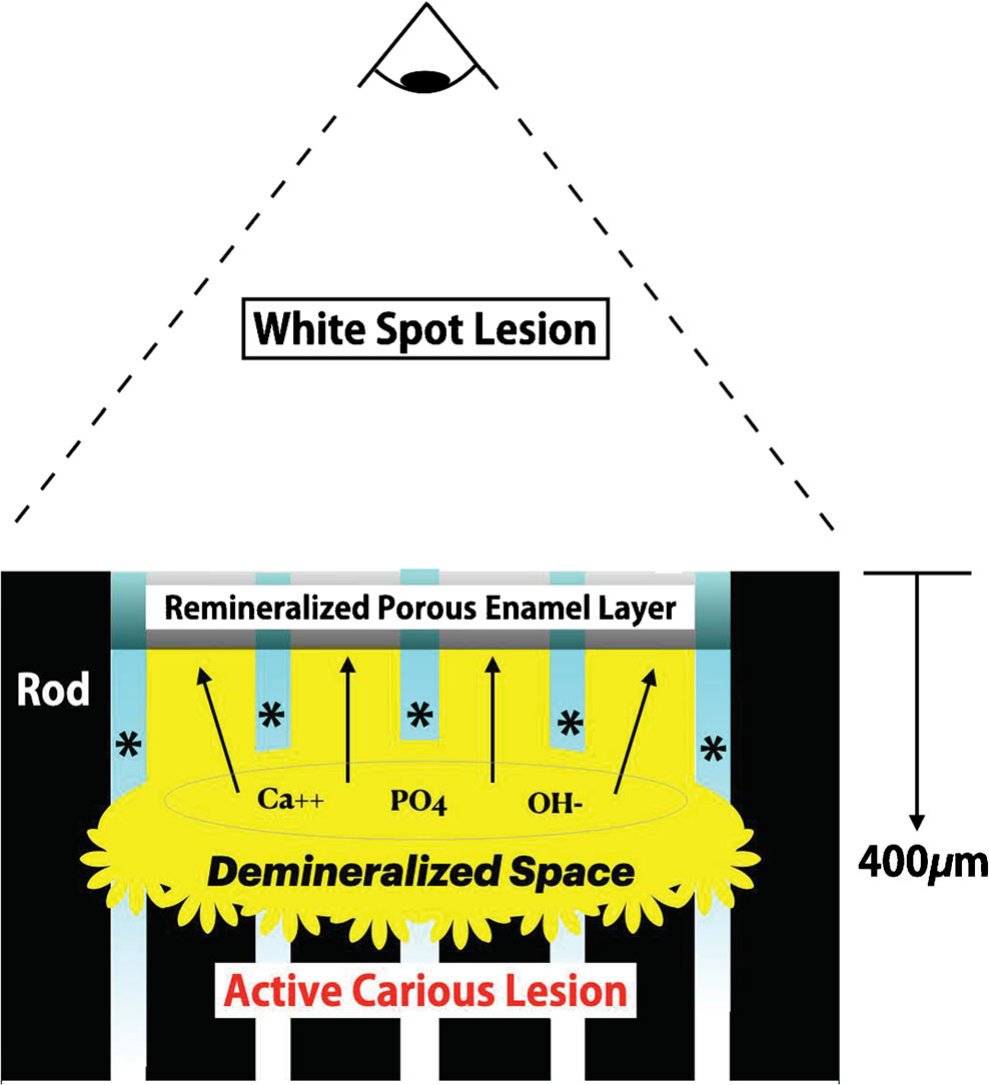
In a 2D section, a WSL is viewed from above as a white discoloration surrounded by intact enamel that reveals the subsurface location of a demineralized space >400μm in depth. It is hypothesized that mineralized inter-rod substance (asterisk) is less susceptible to demineralization because of natural FA formation via intercrystalline percolation. As the FA gradient decreased with depth, the active carious lesions progress laterally and coalesce to form a demineralization space. The lesion is invisible until it reaches a depth of ~400μm when light diffraction is adequate for the demineralized space to be visualized as a subsurface WSL. Evidence suggests the superficial remineralized porous enamel layer is a product of residual enamel not destroyed by the initial surface demineralization that is supplemented by remineralization via calcium (Ca^++^), PO_4_, and OH^−^ moving toward the surface (black arrows). The source of the ions for remineralization is from HA destruction by the active carious lesion at the base of the demineralized space. See text for details

Decalcification and remineralization of all mineralized tissues contribute directly or indirectly to calcium homeostasis. However, percolation of calcium ions from nonliving enamel is via dentin [1]. Caries is initiated as an enamel subsurface demineralization that is morphologically similar to catabolic bone modeling on periosteal surfaces, particularly renal osteodystrophy (ROD) [27, 28]. Furthermore, the intracortical resorption of metacarpal bones with advanced ROD is reminiscent of white spot lesions (WSLs) which are carious lesions with a superficial veneer of enamel (Fig. 2) [29•]. More persistent ROD associated with deep penetration of the cortical bone along phalanges is like cavitated caries. In addition, noncavitated caries are morphologically similar to resorption cavities during bone remodeling [2, 3, 30] or the osseous radiolucencies defined as traumatic cysts [31].

Caries is responsible for many periapical bone infections of the jaws (prevalence >62%) [32], while periodontitis, an alveolar bone infection, results in a loss of bone support (prevalence 11%) [33]. OP is a metabolic bone disease rather than an infection. There is no clear relationship between OP and periodontal bone loss [34]. Caries is a dental (odontogenic) infection that may result in pulp degeneration and spread into the supporting jaw bone [20, 21•, 22, 35]. These are typically polymicrobial infections involving various facultative and strict anaerobes [35].

### Osteomyelitis

Caries-related bone infections are a form of osteomyelitis (OM), i.e., inflammation of bone and marrow tissue. Odontogenic bacterial or fungal infections may spread to deep fascial spaces and can lead to septicemia if not effectively managed. Efficient treatment is to eliminate the source of the infection such as a carious tooth; antibiotics are used as adjunctive therapy [20, 21•, 22]. A necrotic pulp that is devitalized by caries results in periapical osseous infections with the microbes from the carious lesion. Advancement of the infection is similar to caries attacking vital dentin. Bacteria laden biofilms support infection within the dead bone [36] which is a particular problem for orthopedic implant failure [37]. The bacteria feed off the dead tissue in an anaerobic environment. Because the infected tissue tends to be avascular, OM is difficult to resolve with antibiotics [36]. Similar to the surgical excision of active caries with operative dentistry [38, 39], osseous resection is often required to resolve OM [13, 14, 40].

OM of the jaws may be caused by dental sepsis (caries), oral surgical procedures, or facial fracture (trauma). The pathology may be an acute abscess, but most OM of the jaws is diagnosed from routine dental radiographs. Asymptomatic pathology is usually a relatively innocuous, localized lesion that grows slowly. Depending on the virility of the infection, OM may appear to be an area of osteolysis or condensing osteitis in a radiograph [41]. Endodontics and restorative dentistry are usually effective for controlling the source of the infection. Well-managed extensive periapical lesions heal spontaneously. An infection that fails to respond to conservative therapy and antibiotics may be a relatively avascular lesion. Dead bone supports an infection much like cavitated caries, so surgical resection is required [42]. Caries are currently managed with expensive surgical and restorative procedures, i.e., operative, endodontic, and prosthodontic dentistry. However, advances in early caries detection and remineralization therapeutics may redirect dental practice toward prevention. Surgical correction of caries to prevent bone infections and their serious sequelae is neither efficient nor cost-effective.

OM of dental origin is usually an opportunistic infection caused by a mixture of *Alpha hemolytic streptococci* and anaerobic species from the oral cavity [43]. Because of skin exposure, OM of the traumatized jaws or long bones are typically *Staphylococcus aureus* infections [44]. The mandible is affected more commonly than the maxilla probably because the latter has greater vascularity, relatively thin cortical plates, and fewer large medullary spaces. Decreased vascularity of mandibular bone renders it more susceptible to medication-related osteonecrosis of the jaw (MRONJ) associated with resorption-inhibiting drugs [45]. A history of antiresorptive drug therapy may be associated with compromised orthodontic outcomes [46].

Periodontal bone infections are a different manifestation of oral OM. *Porphyromonas gingivalis* is a gram-negative anaerobic pathogen found in subgingival plaque associated with progressive periodontitis [47]. Changes in the oral environment such as orthodontic treatment with fixed appliances and aligners may alter the microbiome of the oral cavity [48]. Significant increases are noted in anaerobic and facultative anaerobes in both plaque and saliva. Fixed orthodontic appliances induced measurable changes in the oral microbiome including a relative abundance of obligate anaerobes that are periodontal pathogens [49]. For a healthy dentition, fixed orthodontic treatment has a transient effect on periodontal health that is ameliorated after removal of the appliances [50], but enamel decalcifications due to high levels of cariogenic bacteria are common [51]. Orthodontics may alter the oral microbiome, but the periodontal effects after treatment are reversible [52].

### Caries

Caries is a unique infection of mineralized tissue caused by biofilm (plaque) containing primarily two species of virulent bacteria: *streptococcus* and *lactobacilli* [4, 5]. Fermentation of dietary carbohydrates produces lactic acid which demineralizes the enamel surface at the plaque interface. A complex demineralization and remineralization cycle related to diet results in a subsurface carious lesion that is covered with what appears to be an intact enamel surface [53]. The pathogenesis of these unusual lesions is clarified with quantitative polarized light analysis of histologic sections [54•] and scanning electron [55•] microscopic studies of lesions induced by 1–4 weeks at the interface of specifically designed plaque-filled spaces beneath orthodontic bands. The device is a simulation of band with intact (control) enamel surfaces which show some demineralization within 10μm of the oral surface [54•]. In the absence of plaque, these potential lesions are limited by saliva-mediated remineralization [6–8]. However, constant plaque contact results in universal enlargement of intercrystalline spaces between the enamel rods. Some enamel pores increase to ~1μm in diameter [55•] which allows nutritive carbohydrates to diffuse into the subsurface lesion (demineralized space). An active subsurface caries process deepens the lesion by eroding Ca^++^ and PO^3−^ at the enamel interface (Fig. 2). These ions escape into saliva through the enamel pores as the lesion advances beneath a largely intact enamel surface that is up to ~9μm in thickness [54•]. The outer visually intact surface is composed of original enamel that was resistant to demineralization that has been supplemented by remineralization of interprismatic substance [56] with Ca^++^ and PO_4_^3−^ produced by active caries in the subsurface lesion. Intra-enamel caries is invisible until the lesions reach a depth of ~400μm. Backscattering of incident light by the subsurface demineralized region is directly related to lesion depth [57]. When a lesion is 400μm deep, the backscatter effect is sufficient to visibly detect the decalcified lesion as a white spot [58, 59]. Figure 2 is a summary of the unique pathogenesis of invisible subsurface lesions that evolve into white spots and may eventually cavitate.

Dental caries is a common oral disease with a complex epidemiology relative to lifestyle, nutrition, and hygiene proficiency. For instance, the disease is more prevalent in cultures consuming carbonated beverages (soft drinks) [60]. Obese children are particularly prone to caries because of habitual carbohydrate-rich snacks between meals [61]. Pits, fissures, restoration margins, and other continuity defects in enamel are predisposed to caries [4, 62], but smooth surfaces are at risk if they are covered with plaque [6, 7, 54•, 55•]. An acidic pH at the enamel surface facilitates the demineralization process [5]. The pH in plaque is depressed for about an hour after each ingestion of acidic food [63], beverages [64], drugs [65, 66], mouthwash [67], and fermentable carbohydrates [4, 5]. Dietary acidity has an accumulative demineralization effect that facilitates caries. Carious lesions in nonvital enamel require the nourishment of sugar or other fermentable carbohydrates from the diet (Fig. 2). However, once the virulent organisms penetrate in living dentin, they are no longer dependent on the diet for nutrition because they live off the tissue they destroy [5]. The odontoblasts in the pulp have cellular processes that penetrate the dentin to the DEJ. When the carious infection enters dentin, there may be an uncomfortable sensation, but no obvious symptoms are apparent until the dental pulp is threatened or infected [1]. Odontoblasts can sense the inflammatory reaction of approaching caries and then resist the carious attack by forming secondary dentin to protect the pulp from infection [12].

### Incipient Lesions

Pellicle formed by salivary proteins coats all intraoral mineralized tissue surfaces. It is a double-edged sword with respect to caries protection. Pellicle protects enamel surfaces from demineralization by acidic foods and saliva [54•], but it is also the foundation for cariogenic biofilm (plaque) that adheres to all mineralized tissues in the oral cavity [68–71]. It is important to reiterate that intra-enamel caries that develop into WSLs are not visible until the noncavitated subsurface porosity reaches a depth of about 400μm (Fig. 2) [57]. Reliable detection of invisible caries in lesions as shallow as 20μm would be valuable for assessing the risk of fixed appliances prior to orthodontic treatment. As subsequently discussed, multiple available and developmental technologies are being evaluated in this regard [4, 5, 72]. However, there is no innocuous method available for detecting invisible carious lesions before characteristic white spots become visible. Preventative measures such as photodynamic control of cariogenic bacteria [73], F- varnish [74], and particularly remineralization are more effective for managing shallow lesions (<20μm) [75]. A lack of proven technology to detect and assess early carious lesions (say <100μm) in enamel is a major deficit in clinical practice because caries intervention and remineralization technology should only be used when indicated. For example, fluoride varnish is a risk for allergic reactions [76]. The risk and expense for prevention and remineralization procedures in patients with no signs or symptoms of caries is only justified by a reliable diagnosis of invisible intra-enamel lesions.

## Caries Classification

The International Caries Detection and Assessment System (ICDAS) established an integrated system for measuring caries that was designed to facilitate clinical research, particularly epidemiology [77]. A seven-point ordinal scale scores sites from sound enamel to extensive cavitation. It is an appropriate method for research but too complex and time-consuming for routine clinical practice. The American Dental Association (ADA) followed up with a relatively simple classification for caries that distinguishes between cavitated and noncavitated lesions [78]. Advanced (cavitated) lesions, root surface caries, and pit and fissure defects are common clinical problems that have important health implications; however, that subject is outside the scope for this review. The current objective is to compare sound enamel surfaces to incipient caries (invisible or white spots) within the context of bone physiology, calcium metabolism, and biomechanics [79]. WSLs are noncavitated, potentially reversible carious lesions that are entirely within enamel [80, 81]. The pathophysiology of interest is comparison of sound enamel to an initial lesion [82]. In diagnosing an incipient lesion (invisible or white spot), it is important to score the defect as either an active or arrested lesion [83]. Sound enamel has normal translucency with a smooth, hard surface. A slight demineralization of an enamel surface (say <100μm deep) is an invisible carious lesion that tends to be slightly more opaque with a loss of enamel luster [83]. Under routine clinical conditions, subsurface caries <400μm deep are invisible [57]. Intra-enamel lesions are typically covered with plaque which is easily disclosed with stain. A plaque-covered enamel surface may feel increasingly rough as the tip of an explorer engages pores that support a subsurface active lesion. There are no clinical methods available for reliably detecting invisible lesions per se*.* Once intra-enamel caries reaches a depth of about 400μm, they become visible as white spots [57]. Current diagnostic technologies such as electrical conductance and quantitative laser fluorescence (QLF) [84] have about the same sensitivity (~400μm) as visual inspection, so neither method offers any real advantage for reliably detecting invisible caries. Cross-polarization optical coherence tomography is a useful research tool for imaging the internal structure of white spots [82], but the clinical utility of this method has not been demonstrated.

If the cariogenic microbes driving an enamel infection are devitalized, the lesion is arrested (Fig. 1), and the carious front remineralizes to become densely mineralized with an off-color hue (white, brown, or black) that is shiny, hard, and smooth [83]. Arresting carious lesions with the medical disinfectant silver nitrate (AgNO_3_) which was referred to as “Howe’s solution” was a common clinical procedure in the nineteenth century [85]. After sodium fluoride (NaF) was established as an anti-caries agent in the twentieth century [86], an improved variation of the Howe’s procedure was to disinfect the lesion with AgNO_3_ and then apply NaF to achieve a dense caries resistant layer via remineralization. In current practice, 25% AgNO_3_ nitrate followed by 5% Na fluoride or 38% Ag diamine fluoride are capable of arresting caries and promoting remineralization [85–87]. Unfortunately, the Ag component of the disinfectant permanently stains the arrested lesion black. That may be an acceptable outcome for posterior teeth in aging and young patients, but it is esthetically unacceptable for the dental surfaces that are exposed when smiling.

## Detection and Assessment of White Spots

WSLs are focal subsurface decalcifications in enamel that are common iatrogenic problems associated with extended plaque retention (Figs. 1 and 2) [88,89]. Any irregularity along an enamel surface (fracture, chip, crack, or bonded attachment) may retain plaque and be cariogenic. In addition, plaque adhering to exposed oral bone sequestra inflame the surrounding mucosa [46, 90–92]. From a physiologic perspective, WSLs are localized pathologic processes (caries) that may or may not be active [83]. From an osseous perspective, a WSL is analogous to some manifestations of negative calcium balance, i.e., accelerated bone remodeling [30] or relatively quiescent bone cysts [31]. It is clear that bones and teeth are susceptible to severe destruction in the presence of sustained demineralization. During routine clinical examination, WSLs are commonly associated with obscure (interproximal) cavitated lesions [80] particularly if there is extensive plaque accumulation near gingival margins [4, 5]. However, WSLs can occur in any protected area that is not cleaned by the mechanical action of soft tissue, inserting and removing a removable appliance [93], or oral hygiene procedures [59, 73, 89].

There are no documented reports for decalcifications with removable aligner treatment [94], which is curious because numerous online blogs and practitioner websites suggest it is a serious problem for patients with poor hygiene and a cariogenic diet [95]. The principal concern is plaque accumulation both on teeth and inside the aligners [71]. In addition, an aligner may inhibit the natural buffering and cleansing effect of saliva [4, 5, 88]. Risky behavior is a patient with poor hygiene who consumes fermentable carbohydrates [4, 5, 88], prior to placing an aligner. An environment is created for WSLs or generalized decalcifications concentrated at the cusp tips [95]. Biofilm can also form inside aligners and on underlying enamel surfaces particularly if there are bonded attachments [71, 95]. Fermentable carbohydrates in the diet are serious concerns for aligner treatment. Reportedly teeth covered by aligners are susceptible to generalized decalcifications [60, 61, 95]. Plaque removal from enamel surfaces and around attachments is heavily dependent on hygiene measures. Furthermore, an aligner shields teeth from the mechanical action of the soft tissues and may inhibit buffering and remineralization by saliva [4, 5, 95]. Although clinicians may have observed these problems, it is important to note that no documented reports are published [94]. Similar to removable functional appliances [93], aligners may mechanically remove plaque from tooth surfaces when they are inserted, worn, and removed. Fixed appliances are clearly the most important risk factor for WSLs because the prominent devices and bonding materials promote retention of cariogenic plaque [89, 96].

## Etiology of WSLs

The etiology for WSLs is cyclic periods of demineralization due to acid attack after fermentable carbohydrate consumption [4, 88] followed by remineralization during the between meal/snack resting period [81]. The initial invisible foci are propagated by cariogenic bacteria that produce and invade defects in the enamel surface created by demineralization of enamel rods (Fig. 1) [4–7]. Within individual carious sites, anaerobic metabolism of cariogenic bacteria within biofilm produces primarily lactic acid from dietary sugar. Once early subsurface lesions are established, the cariogenic bacteria and their supporting biofilm are resistant to mechanical abrasion; however, a lesion within enamel remains dependent on fermentable carbohydrates from the diet for nourishment [4–7]. As the demineralized foci deepen >20 μm, they may coalesce into a large subsurface lesion with a front of cariogenic bacteria that continue the caries process (Fig. 2) [53, 54•]. The calcium and phosphate ions freed by the demineralization process at the carious front move away from the active front of the lesion and supplement the porous enamel layer over a WSL (Fig. 2). Remineralization occurs in the inter-rod sheath, along the residual enamel veneer, or by partially filling-in enamel pores [53, 54•, 55•]. Thus, WSLs evolve a distinct morphology: hard enamel surface with an underlying demineralized cavity that appears white relative to the surrounding sound enamel [89, 96, 97]. The internal disease process is elucidated with polarized microscopy and micro-radiographic images of tooth sections that are cut perpendicular to the lesion [54•, 98, 99]. Raman microscopy is also capable of detailed imaging of a lesion [29]. Natural and induced WSLs show four histologic zones: (1) intact surface layer (ISL), (2) body of the demineralized lesion (white opacity), (3) dark zone (base of the demineralized area), and (4) translucent zone (advancing front of the lesion) [29•, 54•]. In effect, a WSL is an activation (A) of microbial resorption (**R**) in enamel that bears similarities to an osteoclast front resorbing the interior aspect of cortical bone during catabolic modeling (A—>**R**) [27]. Reversal of R leads to formation (F) during bone remodeling (A➔R➔F), but coupling may be arrested so the initial phase of remodeling (A—>**R**) produces a residual resorption cavity [2, 3]. Furthermore, the acidic front of the cariogenic microbes in a WSL is similar to the clear “sealing zone” of osteoclasts resorbing bone [100].

The inter-crystalline spaces within the rod sheath are larger and more permeable than the rods themselves [55•]. Morphology is consistent with increased fluid flow (percolation) to support turnover of HA via demineralization and remineralization. This mineral exchange mechanism provides the opportunity to introduce F^−^ deep in the enamel structure to form FA. Forming FA deep in the mineral component of the rod sheath has important physiologic implications. FA in the inter-rod substance may be the reason that acid etching procedures show that the rod sheath is usually more resistant to demineralization (Fig. 1) [101, 102]. The clinical benefit for this aspect of differential etching is the type 1 etching pattern with deep surface recesses which is favorable for bonding to enamel. The unfavorable type 3 etching pattern (relatively flat surface) may reflect a patient history of inadequate exposure to F^−^. Furthermore, the retained rod sheath after the rods are demineralized provides a substrate for remineralization of the superficial enamel layer which explains the mechanism for producing a WSL. A history of adequate F^−^ to produce FA by remineralization deep in the rod sheath mineral fraction may differentiate enamel surfaces predisposed to WSLs rather than cavitated caries. Thus, WSLs are characteristic of plaque-coated smooth enamel surfaces [88, 89, 96, 103]. However, demineralization-resistant inter-rod substance of subjects with a history of F^−^ exposure may be an important aspect of the specific pathogenesis [1]. Furthermore, enamel is an anisotropic material with a tough network of interconnected fibrous tissue, i.e., inter crystalline substance or matrix [104]. Similar to bone, enamel can maintain its structural integrity despite considerable internal porosity [53, 54•, 55•].

When the demineralization process associated with the action of plaque acid overwhelms the balancing remineralization capability of saliva and hygiene, caries is active, and there is a net loss of tooth mineral over time (Fig. 1) [103]. For over 50 years, it has been known that early (incipient) caries are not clinically perceptible, but the lesions within enamel can be detected with histologic sections [54•]. Active caries progressively increases the depth of the demineralized zone in the direction of the DEJ. Once the subsurface zone of enamel porosity reaches a depth of about 400μm, air drying of the enamel surface for ~5 s reveals a visually perceptible white spot lesion [54•, 77]. The width of the subsurface demineralized zone (Fig. 2) increases light scattering [57, 105] which disrupts the normally anisotropic translucence of enamel [106]. It is important to reemphasize in this context the lack of a reliable clinical procedure to detect and assess invisible subsurface carious lesions. This problem has been recognized for some time in restorative dentistry. Considerable research and development is focused on determining which white spots and invisible subsurface lesions are sites of active caries because no treatment is indicated for inactive lesions [107, 108]. On the other hand, for orthodontics, it is important to detect and manage all subsurface enamel demineralizations prior to bonding fixed appliances. The rapid deterioration of susceptible patients with labial [54•, 59, 89] and to a lesser extend lingual [109] appliances suggests a heavy plaque load associated with poor hygiene potentiates active lesions and reestablishes active caries in previously inactive sites. In contrast to restorative dentistry, orthodontics requires a comprehensive method for detecting and managing *all subsurface demineralizations.*

Invisible caries is defined as protected (subsurface) decalcified sites (<400μm deep) that are resistant to mechanical abrasion, dental hygiene, or professional prophylaxis (Fig. 1) [82, 96]. Polishing enamel surfaces with pumice prior to bonding orthodontic appliances removes both plaque and the protective proteins of the salivary pellicle. Noncavitated WSLs (>400μm deep) can occur within 4 weeks in a defined, plaque-filled space under an orthodontic band [54•, 55•]. Routine follow-up of active treatment patients may document extensive WSLs within 12months after bonding brackets [89]; but particularly susceptible patients are affected in only 3–6month. It is hypothesized that high-risk patients had undetected subsurface lesions prior to the start of treatment. Retained plaque around orthodontic brackets accelerates active caries and reinoculates previously inactive lesions. Patients with poor hygiene and a carbohydrate-rich diet may experience WSLs within weeks after the start of active treatment [54•].

Orthodontically induced WSLs are a major problem worldwide [54•, 59, 89, 109]. It is not only an esthetic issue because WSLs may progress to serious cavitated caries during or after active treatment [89]. Distinguishing active from inactive lesions is a valid strategy for restorative dentistry, but all enamel surfaces damaged by caries are a concern for orthodontics because caries can be reinoculated by retained plaque. Effective pretreatment and progress screening of orthodontics patients is critical for the management of invisible caries prior to forming WSLs. Multiple new technologies are available or are in development for detecting incipient caries: fiber-optic trans-illumination, quantitative light-induced fluorescence (QLF), digital image fiber-optic trans-illumination, electrical conductance measurement, digital subtraction radiography, laser fluorescence measurement, and ultrasound caries detection [97]. In addition, optical coherence tomography systems are effective for noninvasive assessment of hard dental tissues [72, 98]. The emphasis for most new device development is on pits, fissures, and interproximal caries [99]. None of the physical methods currently available are capable of detecting shallow (<400μm deep), invisible lesions on smooth enamel surfaces.

A safe, rapid, and reliable method for detecting early invisible carious lesions is badly needed. If teeth with compromised enamel are detected prior to bonding fixed appliances, pretreatment preventative and repair procedures can be accomplished prior to bonding fixed appliances. Orthodontics with fixed appliances on labial surfaces is a concern for patients predisposed to smooth surface caries because the enamel is relatively thin in the cervical area gingival to the bonded brackets [1]. Cavitated caries can rapidly penetrate the pulp. In addition, a simple method with adequate sensitivity for detecting invisible caries would be very useful during orthodontic treatment to monitor high-risk zones. Early detection of invisible lesions is critical for the timely intervention with preventive and corrective measures to avoid WSLs. For some uncooperative patients, discontinuing elective treatment may be indicated.

## Emerging Technologies

Three emerging technologies show promise. First, the CALCIVIS® Imaging System employs a bioluminescence technology to detect free calcium ions emitted by active carious lesions [107]. This system measures the level of caries activity [108], as a function of free calcium ions within WSLs, but it is “not a caries detection device.” [107] The application of CALCIVIS® for detecting and managing clinically invisible incipient lesions (<400μm) has not been demonstrated. Second, cationic fluorescein-labeled starch nanoparticles have been developed in vitro for the assessment of caries activity [110]. This system may prove useful for detecting active subsurface caries, but it does not appear suitable for detecting *all* incipient lesions in the enamel. Thus, the method is not suitable for the thorough enamel evaluation required prior to orthodontics. Finally, a group in Australia with extensive experience in molar-incisor hypomineralization [111–113] developed a hydroxyapatite-binding porosity probe based on bovine hemoglobin that shows promise for detecting shallow subsurface carious lesions down to depth of ~30 microns [114]. Colorimetric detection of invisible lesions is via HA-binding proteins coupled to intensely colored dyes. The technology is relatively simple, safe, and intuitive for a dentist in a routine clinical setting, so it shows promise for both early detection and activity monitoring.

Collectively, these emerging technologies provide a perspective for detection and assessment of early-stage carious lesions. All the technologies require approval for use in human subjects as well as clinical validation for sensitivity and specificity. For orthodontic purposes, it is important to disclose all subsurface porosity and then attempt to remineralize it prior to orthodontic treatment. Periodic evaluation during active treatment should emphasize detection of active carious lesions on all exposed enamel surfaces.

## Treatment of WSLs

Prevention is the most effective and efficient management for WSLs. Pretreatment education and motivational training in oral hygiene procedures with a F^−^ containing toothpaste and diet control are indicated prior to elective treatment such as extensive restorations or orthodontics with fixed appliances [96]. Reasonable adherence to plaque and diet control is usually all that is needed to prevent decalcifications and caries. However, some potential patients fail to comply, and continued emphasis on preventative measures is counterproductive. A common scenario is parents who are motivated for treatment of their children who are not motivated and may even be defiant. Marginal or poor compliance with diet and/or hygiene by a potential patient is a contraindication for elective treatment [58, 89]. In the absence of an effective diagnostic test for invisible caries, a plaque index is currently the best indicator for patients at high risk for WSLs. If a patient fails to demonstrate adequate plaque control, extensive restorative dentistry or fixed orthodontic appliances are contraindicated. For resolving malocclusion for patients with questionable hygiene, it may be appropriate to pursue limited treatment with lingual arches, removable appliances, or aligners [93, 94]. If plaque retention and gingivitis are noted during recall visits, additional brushing instructions and plaque control technology such as chlorhexidine, microtome dynamics, and photodynamic therapy may be needed [49, 73]. Archwires can be removed to allow better access for hygiene. When WSLs are noted, that is a clear indication that plaque control is inadequate. It is best to discontinue high-risk treatment and pursue other options rather than seriously damage the dentition [89, 96].

The importance of F^−^ for preventing WSLs has resulted in numerous products: mouthwash, gels, toothpaste, varnishes, bracket bonding agents, and elastomers [74–76, 88, 96]. F^−^ can control caries by inhibitory effects on cariogenic microbes which may result in an arrest of the active caries process. Moderate amounts of F^−^ are effective for remineralization. For an optimal fluoride effect, mouth washes and toothpastes should be applied daily. Fluoride varnishes require application from 2 to 4 times per year [96]. Enamel sealing resin as well as bracket bonding materials and elastomers that release F^−^ have been less successful because there is an inadequate release of F^−^ and/or the mechanical properties of the materials are compromised.

In the absence of cavitation, WSLs are potentially reversible [4, 5]. Natural remineralization with salivary ions is the preferred method for resolving WSLs. The first step for this approach is to establish good oral hygiene and provide regular professional prophylaxis at 3–6-month intervals. With good plaque and diet control, the carious lesion at the base of a WSL is deprived of nourishment, so the lesion is arrested [83, 108]. Then Ca^++^ and PO_4_^3−^ from the saliva can penetrate through pores in the enamel veneer to remineralize a WSL. Modest daily exposure to F^−^ is advantageous. It is important to avoid large accumulative doses of F^−^ with topical application, rinses, toothpaste, mouthwash, gels, and/or varnish. Forming a dense, less porous layer of FA along the surface enamel may decreases the flow of Ca^++^ and PO_4_^3−^ to the subsurface WSL. Tooth bleaching whitens the teeth and thereby masks WSLs [115], but there is no effect on remineralization [116, 117]. Overall, correction of WSLs is a challenging clinical problem because the rigorous hygiene required is unrealistic for most patients with a history of poor compliance.

Casein derivatives were developed to penetrate the enamel barrier and increase the supply of Ca^++^ and PO_4_^3−^ to enhance remineralization [116, 117]. Casein phosphopeptides-amorphous calcium phosphate (CPP-ACP) is a mineralization agent produced from milk that helps ameliorate WSLs. F^−^ is a helpful adjunct in modest amounts, so it is added to the casein remineralization paste and dental varnish. These products are popular in clinical practice, and many offices provide the paste at no additional fee. That may be a wise strategy for limiting liability in refractory patients. However, routine remineralization of WSLs remains elusive. A systematic review published in 2016, reported good oral hygiene with high-fluoride tooth paste, is comparable in effectiveness to F^−^ supplemented casein derivatives for prevention of new WSLs [96]. For treatment of WSLs, neither MI Paste Plus® (GC America, Alsip, Ill) nor PreviDent® (Colgate Oral Pharmaceuticals, New York, NY) F^−^ varnish was more effective than routine oral hygiene procedures for improving WSLs in an 8-week clinical trial after orthodontic treatment [118].

Preventing WSLs is best accomplished with a careful history. Children may have been treated with nonsurgical caries management techniques [119–121], so the risk of decalcifications may not be obvious. Lack of active caries does not preclude inactive enamel lesions that can be reinoculated due to inadequate oral hygiene measures. If prevention fails, all the restorative measures for correcting WSLs have limitations. Resin infiltration improves enamel esthetics but does not remove WSLs [122]. The most reliable restorative procedure for removing WSLs is microabrasion to remove the outer enamel barrier to allow the internal decalcification to remineralize with salivary ions [123]. Fluoride supplemented, casein derivative paste may be more effective after microabrasion because the enamel barrier is removed [123, 124]. The obvious concern with microabrasion is the net loss of enamel, but compared to veneer restorations, it is considerably more conservative, cost-effective, and less prone to long-term problems [125–127].

## Removable Lingual Arch

Enamel decalcifications have been a risk in orthodontics since the initiation of modern fixed appliances in the early twentieth century [128–130]. The generalized decalcification tendency long noted with banded appliances is exacerbated by loose bands as well as exposed enamel between the inferior border of a band and the gingival margin. Gorelick et al. (1982) [131] coined the term “white spots” to define the orthodontic lesions that are characteristic for both banded and bonded teeth. WSLs are very common for bonded brackets (prevalence >36% in patients with poor hygiene and a cariogenic diet) [89]. Dr. John Valentine Mershon (1867–1953) was a 1908 graduate of the Angle School of Orthodontia. He trained in an era when heavy forces to move teeth were applied to the labial surfaces of the dentition [132]. Dr. Mershon was concerned that large applied loads were not physiologic, so he invented or at least popularized the use of a removable lingual arch (RLA). A RLA is readily adjustable to “gently nudge teeth into more desired positions.” An important serendipitous benefit of the appliance is that it is much less prone to plaque retention. Decalcifications have not been reported for the lingual surfaces of teeth treated with labial appliances [89, 131]. Thus, a RLA is largely plaque free, and it applies forces on the self-cleansing lingual surfaces [109], so decalcification is unlikely.

Use of bonded brackets during mixed dentition treatment is associated with long treatment times, compromised outcomes, and serious enamel decalcifications [89, 133]. Control of decalcifications (WSLs) was not a stated goal for Dr. Mershon, but it is important to note that there are no decalcification reports associated with the use RLAs in over a century of use. The RLA is useful for maintaining and correcting arch widths during active treatment [134, 135]. In addition, RLAs have proven to be particularly efficient for mixed dentition treatment in the lower arch. They are effective for space management, arch development, and alignment during the juvenile years. The only appreciable risk for decalcification is loose bands on the first molars. When using RLAs for lengthy mixed dentition treatment, it is important to utilize glass ionomer cement for first molar bands, ask the patient and parent to regularly monitor band retention, and clinically test band retention at each treatment visit. A RLA is very efficient for low-risk mixed dentition treatment for class I crowded malocclusions [136], but it is also useful for class II (retrognathic) malocclusions [137]. Compared to labial fixed appliances, RLA treatment presents much less risk for iatrogenic decalcifications and WSLs [59, 89, 96, 131].

## Conclusions

WSLs are mineralized tissue pathophysiology that is often obscure to clinicians. Understanding the etiology, progression, arrest, and remineralization of WSLs is crucial for ideal orthodontic practice. Efficient WSL management begins with patients demonstrating good oral hygiene and fluoridated toothpaste before starting elective treatment. The physiologic rationale is that teeth are perfused with ion-rich fluid from the saliva which is capable of substituting F^−^ deep into the HA structure to resist demineralization. During active treatment, hygiene with fluoridated toothpaste should be enforced at each appointment. If WSLs are detected, the braces should be removed as soon as possible to initiate a follow-up remineralization program that focuses on plaque control with a fluoridated toothpaste. It is important to avoid excessive F^−^ during remineralization because a very dense enamel veneer on a WSL can inhibit the percolation of calcium and phosphate ions into the subsurface lesion. To prevent WSLs, there is a glaring need for a simple clinical technology to detect shallow invisible caries. Remineralization procedures prior to orthodontic treatment and effective monitoring of enamel deterioration during treatment would greatly enhance the clinical prevention and control of WSLs.
